# Impact of VEGF, VEGFR, PDGFR, HIF and ERCC1 gene polymorphisms on thymic malignancies outcome after thymectomy

**DOI:** 10.18632/oncotarget.4191

**Published:** 2015-06-08

**Authors:** Rossana Berardi, Alessandro Brunelli, Silvia Pagliaretta, Vittorio Paolucci, Alessandro Conti, Gaia Goteri, Majed Refai, Cecilia Pompili, Giulia Marcantognini, Francesca Morgese, Zelmira Ballatore, Agnese Savini, Mariagrazia De Lisa, Miriam Caramanti, Matteo Santoni, Antonio Zizzi, Francesco Piva, Paola Mazzanti, Azzurra Onofri, Armando Sabbatini, Marina Scarpelli, Stefano Cascinu

**Affiliations:** ^1^ Medical Oncology, Università Politecnica delle Marche, Azienda Ospedaliero-Universitaria Ospedali Riuniti Umberto I – GM Lancisi – G Salesi, Italy; ^2^ Department of Thoracic Surgery, St. James's University Hospital, Leeds, West Yorkshire, United Kingdom; ^3^ Thoracic Surgery, Azienda Ospedaliero-Universitaria Ospedali Riuniti Umberto I – GM Lancisi – G Salesi, Italy; ^4^ Urology, Università Politecnica delle Marche, Ancona, Italy; ^5^ Section of Pathological Anatomy and Histopathology, Università Politecnica delle Marche, Ancona, Italy; ^6^ Department of Specialistic Clinical and Odontostomatological Sciences, Università Politecnica delle Marche, Ancona, Italy

**Keywords:** angiogenesis, prognosis, single nucleotide polymorphism, thymic epithelial tumor, tumor risk

## Abstract

We aimed to analyze genotypes of VEGF-A, VEGFR2, Flt4, PDGFRα, HIF-1α and ERCC1 and their correlation with thymic tumor risk and patient outcome.

DNA of 57 consecutive patients (43 thymomas and 14 thymic carcinomas) who underwent total thymectomy at our Institution was extracted from paraffin-embedded tissue. We selected polymorphisms in the following genes:HIF1-α (rs2057482T > C, rs1951795A > C, rs2301113C > A, rs10873142C > T, rs11158358G > C, rs12434438G > A, rs11549465C > T, rs11549467G > A), VEGF-A (rs2010963G > C, rs699947A > C), VEGFR-2 (rs2305948C > T, rs1870377T > A), VEGFR-3 (rs307826T > C, rs307821C > A), PDGFR-α (rs35597368C > T) and ERCC1 (rs11615A > G). Gene polymorphisms were determined by Real-Time PCR using TaqMan assays.

As compared to the general population, the allele frequency of PDGFR-α rs35597368T was significantly higher (95% vs. 87%, *p* = 0.036), while the frequency of alleles HIF1-α rs2057482C (78% vs. 90%), rs1951795C (69% vs. 87%), rs2301113A (70% vs. 83%), rs10873142T (70% vs. 87%), rs11158358C (75% vs. 88%), rs12434438A (67% vs. 84%) were significantly lower. VEGFR-3 rs307821C frequency was significantly higher in thymomas vs. thymic carcinomas (79% vs. 72%, *p* = 0.0371). The following factors were significantly correlated with a longer overall survival: VEGFR-3 rs307826C, VEGFR-2 rs1870377A, PDGFR-α rs35597368T/C, HIF1-α rs2301113C, rs2057482C/T, rs1951795C, rs11158358G/C and rs10873142T/C, ERCC1 rs11615A (*p* < 0.05).

Our results suggest, for the first time, that PDGFR-α, HIF-1α and VEGFR-3 SNPs are associated with thymic cancer risk and survival.

## INTRODUCTION

Thymic epithelial tumors (TETs) are rare malignancies with an overall annual incidence of 0.15 per 100.000 inhabitants. Emerging data indicate that thymomas and thymic carcinomas (TC) are distinct entities, characterized by peculiar anatomical and clinical features, biological behavior, gene expression and sequencing data. In addition, intra and intertumor heterogeneity has been reported even in patients with the same histotype. [1–4]

Despite several advances in molecular biology are shedding light onto the variety of genetic aberrations involved in thymic carcinogenesis, our current understanding still remain limited. Angiogenesis has been highlighted as a critical component in this process. Vascular endothelial growth factor-A (VEGF-A) and its receptors (VEGFR1 and VEGFR2) are implicated in regulating physiological and pathological angiogenesis. VEGF-A has been proposed as a proangiogenic and autocrine factor in thymomas and as an immunoregulatory factor in the normal thymus. [5] Both VEGF-A and VEGFRs are overexpressed in TETs compared to normal thymus and seem to be associated with advanced clinical stages, predominantly in TC. [6]

The human *VEGF-A* gene is located on chromosome 6 (6p21.1), whereas *VEGFR2* or *KDR* (kinase insert domain receptor) gene is located in chromosome 4 (4q11-q12). These genes are highly polymorphic in humans, and single nucleotide polymorphisms (SNPs) have been reported. These SNPs may contribute to high variability in *VEGF-A* and expression among tissues as well as influence the circulating plasma VEGF-A concentrations. [7–9]

The frequency of these polymorphisms varies across different populations. Beyond the *VEGF-A* and *KDR*, several genes, such as *VEGFR3* or *Flt4* [10], *Platelet-derived growth factor (PDGF) and PDGF receptor-α (PDGFRα)* [11], *Hypoxia-inducible factor-1α (HIF-1α)* [12], and *Excision repair cross-complementation group 1* (*ERCC1)* [13], have been also associated with tumour angiogenesis and malignant progression.

In this study, we analyzed genotypes of *VEGF-A, KDR, Flt4, PDGFRα, HIF-1α* and *ERCC1* in TETs, aiming to verify whether they correlate with increased tumor risk and/or with the outcome of these patients.

## RESULTS

### Patients characteristics

Fifty-seven patients with TETs were included in this study: 43 (75%) presented with thymoma and 14 (25%) with TC. Clinical characteristics are described in Table 1. Male/female ratio was 31/26, and median age was 60 years (range 21–81y). Eighteen patients (32%) presented with Myasthenia Gravis, while 3 (5%) experienced other syndromes (Lichen ruber planus, Pancytopenia, Coombs-positive Hemolytic anemia and Myositis). Patients underwent a previous biopsy in 50% of cases. Out of the 43 thymomas, 32% were AB, 18% B2, 11% A, 11% B1 and 5% B3, according to WHO classification. Over 61% of the patients had tumors larger than 5 cm. According to the World Health Organization classification, 32% out the 43 thymomas were AB, 18% B2, 11% A, 11% B1 and 5% B3. According to Masaoka-Koga staging, 16%, 32%, 28%, 7%, 4% and 5% of patients presented in stage I, IIA, IIB, III, IVA and IVB, respectively.

**Table 1:** Patients’ characteristics

PARAMETERS	PATIENTS (*N* = 57)
**GENDER**	
Males	26 (46%)
Females	31 (54%)
**DISEASE**	
Thymomas	43 (75%)
Thymic carcinoma	14 (25%)
**AGE AT THE DIAGNOSIS (years)**	
Median (range)	60 (21–81)
**ECOG PERFORMANCE STATUS**	
0	37 (65%)
1	16 (28%)
2	4 (7%)
**CLINICAL SYNDROMES**	
None	36 (63%)
Myasthenia gravis	18 (32%)
Other	3 (5%)
**HISTOLOGY**	
A	6 (11%)
AB	18 (32%)
B1	6 (11%)
B2	10 (17%)
B3	3 (5%)
Carcinoma	14 (24%)
**TUMOR SIZE**	
< 5 cm	21 (37%)
> 5 cm	36 (63%)
**PATHOLOGIC STAGE**	
I	9 (16%)
IIA	21 (37%)
IIB	17 (30%)
III	5 (8%)
IVA	2 (4%)
IVB	3 (5%)

### *Description of polymorphisms,* Hardy–Weinberg equilibrium and linkage disequilibrium

Two SNPs were identified in KDR (VEGFR2) (rs2305948, rs1870377), VEGF-A (rs2010963, rs699947), and Flt-4 (VEGFR3) (rs307821, rs307826). A single SNP was identified in PDGFR-α (rs35597368) and ERCC1 (rs11615) and eight SNPs in HIF1-α (rs2057482, rs1951795, rs2301113, rs10873142, rs11158358, rs12434438, rs11549465, rs11549467). Chromosomal location, position in the gene, base exchange and MAF are shown in Table 2a.

**Table 2a:** Chromosomal location, position in the gene, base exchange and MAF of polymorphism studied group

Gene	ID SNP	Chr	Position CDS	AA Change	Allele Frequencies CEU (HapMap)	
					Main allele	Minor allele
**VEGFA**	rs2010963	6	5′ UTR	-	G = 0.6882	C = 0.3118*
**VEGFA**	rs699947	6	UPSTREAM	-	C = 0.522	A = 0.47
**KDR**	rs2305948	4	c.889 C > T	*p*. V297I	C = 0.920	T = 0.080
**KDR**	rs1870377	4	c.1416A > T	*p*. Q472H	T = 0.7529	A = 0.247*
**Flt4**	rs307821	5	c.3971 G > T	*p*. R1324L	G = 0.9059	T = 0.0941*
**Flt4**	rs307826	5	c.1480 T > C	*p*. T494A	T = 0.898	C = 0.102
**PDGFRα**	rs35597368	4	c.1432T > C	*p*. S478P	T = 0.867	C = 0.133
**HIF1α**	rs2057482	14	3′ UTR	-	C = 0.903	T = 0.097
**HIF1α**	rs1951795	14	INTRONIC	-	C = 0.867	A = 0.133
**HIF1α**	rs2301113	14	INTRONIC	-	A = 0.827	C = 0.173
**HIF1α**	rs10873142	14	INTRONIC	-	T = 0.867	C = 0.133
**HIF1α**	rs11158358	14	INTRONIC	-	C = 0.8824	G = 0.1176*
**HIF1α**	rs12434438	14	INTRONIC	-	A = 0.845	G = 0.155
**HIF1α**	rs11549465	14	c.1744C > T	*p*. P582S	C = 0.925	T = 0.075
**HIF1α**	rs11549467	14	c.1762G > A	*p*. A588T	G = 0.987	A = 0.013
**ERCC1**	rs11615	19	c.354T > C	*p*. N118N	A = 0.642	G = 0.358

**Figure 1:**
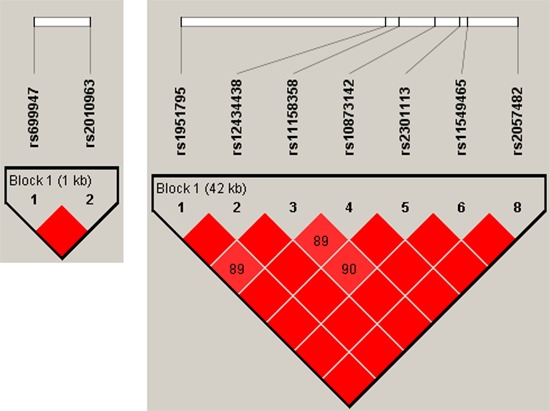
Linkage disequilibrium plot generated by Haploview software Linkage disequilibrium (LD) is displayed as pairwise D’ values. Shading represents the magnitude and significance of pairwise LD, with a red-to-white gradient reflecting higher-to-lower LD values. Red diamond without a number corresponds to D’ values of 1.0.

All SNPs were in Hardy–Weinberg equilibrium (HWE) (Table 2b). The linkage disequilibrium (LD) analysis revealed that VEGFA rs2010963 and rs69947 were in strong LD as well as HIF1-α polymorphisms (Figure 1).

**Table 2b:** Hardy–Weinberg equilibrium of selected SNPs

ID SNP	Chr Position	ObsHET	PredHET	HWpval
**rs699947**	43844367	0.46	0.468	1.0
**rs2010963**	43846328	0.452	0.481	0.7723
**rs11615**	45923653	0.581	0.475	0.1485
**rs35597368**	55139771	0.095	0.091	1.0
**rs1870377**	55972974	0.429	0.387	0.6505
**rs2305948**	55979558	0.175	0.159	1.0
**rs1951795**	62171426	0.444	0.433	1.0
**rs12434438**	62197298	0.46	0.45	1.0
**rs11158358**	62198954	0.444	0.379	0.3255
**rs10873142**	62203462	0.426	0.429	1.0
**rs2301113**	62206548	0.435	0.431	1.0
**rs11549465**	62207557	0.345	0.309	0.7499
**rs11549467**	62207575	0.0	0.0	1.0
**rs2057482**	62213848	0.397	0.354	0.6122
**rs307821**	180030313	0.197	0.203	1.0
**rs307826**	180051003	0.222	0.267	0.3391

### Genotyping and prognostic analyses

This study analyzed the SNP frequency of genes involved in tumor angiogenesis and progression in thymomas and TC compared with general population. All frequencies and genotype distributions are show in Table 3.

**Table 3:** Genotype and allele frequencies of evaluated genes polymorphisms

Gene	SNPs	Allele	Frequencies general population	Frequencies study cohort	*n*. sample	*p*	OddsRatio
VEGFR 2	**rs2305948**	C	92, 00%	90, 35%	57/57	0.6607	1.23
		T	8, 00%	9, 65%			
VEGFR 2	**rs1870377**	T	72, 50%	74, 56%	57/57	0.7243	0.90
		A	27, 50%	25, 44%			
VEGF A	**rs2010963**	G	68, 82%	61, 61%	56/56	0.2571	1.38
		C	31, 18%	38, 39%			
VEGF A	**rs699947**	C	52, 20%	62, 28%	57/57	0.1240	0.66
		A	47, 80%	37, 72%			
VEGFR3	**rs307821**	C	90, 59%	88, 18%	55/55	0.5620	1.29
		A	9, 41%	11, 82%			
VEGFR3	**rs307826**	T	89, 80%	85, 09%	57/57	0.2830	1.54
		C	10, 20%	14, 91%			
PDGFR-α	**rs35597368**	T	86, 70%	94, 74%	57/57	0.0365	0.36
		C	13, 30%	5, 26%			
HIF1-α	**rs2057482**	C	90, 30%	78, 07%	57/57	0.0114	2.61
		T	9, 70%	21, 93%			
HIF1-α	**rs1951795**	C	86, 70%	69, 30%	57/57	0.0015	2.89
		A	13, 30%	30, 70%			
HIF1-α	**rs2301113**	A	82, 70%	69, 64%	56/56	0.0218	2.08
		C	17, 30%	30, 70%			
HIF1-α	**rs10873142**	T	86, 70%	70, 00%	55/55	0.0026	2.79
		C	13, 30%	30, 00%			
HIF1-α	**rs11158358**	C	88, 24%	75, 44%	57/57	0.0122	2.44
		G	11, 76%	24, 56%			
HIF1-α	**rs12434438**	A	84, 50%	66, 67%	57/57	0.0017	2.73
		G	15, 50%	33, 33%			
HIF1-α	**rs11549465**	C	92, 50%	85, 09%	57/57	0.0761	2.16
		T	7, 50%	14, 91%			
HIF1-α	**rs11549467**	G	98, 70%	100, 00%	57/57	0.2220	0.00
		A	1, 30%	0, 00%			
ERCC1	**rs11615**	A	64, 20%	59, 82%	56/56	0.4996	1.20
		G	35, 80%	40, 18%			

The frequency of PDGFR-α polymorphism rs35597368T was significantly higher in thymomas than for general population (94.7% vs. 86.7%, *p* = 0.036). Otherwise, the frequency of following HIF1-α polymorphisms resulted lower than in general population (*p* < 0.05): rs2057482C (78.1% vs. 90.3%), rs11549465C (85.1% vs. 92.5%), rs1951795C (69.3% vs. 86.7%), rs2301113A (69.6% vs. 82.7%), rs10873142T (70.0% vs. 86.7%), rs11158358C (75.4% vs. 88.2%), rs12434438A (66.7% vs. 84.5%). Furthermore, i VEGFR-3 polymorphism rs307821C frequency was higher in thymoma than in TC (79.5% vs. 72.5%, *p* = 0.037). As regards VEGF-A SNPs, we did not observe significant results.

Median OS was 188.4 months (95%CI 138.8−244.7). At univariate analysis, we analyzed the impact of sex, performance status at diagnosis, presence of myasthenia, tumor size, stage and histologic type on prognosis. Furthermore, patients with lower tumor burden (< 5 cm) had longer overall survival (OS) than those with larger tumors (> 5 cm) (median survivals not reached, *p* = 0.049). The other characteristics did not show a significant correlation with OS.

**Figure 2:**
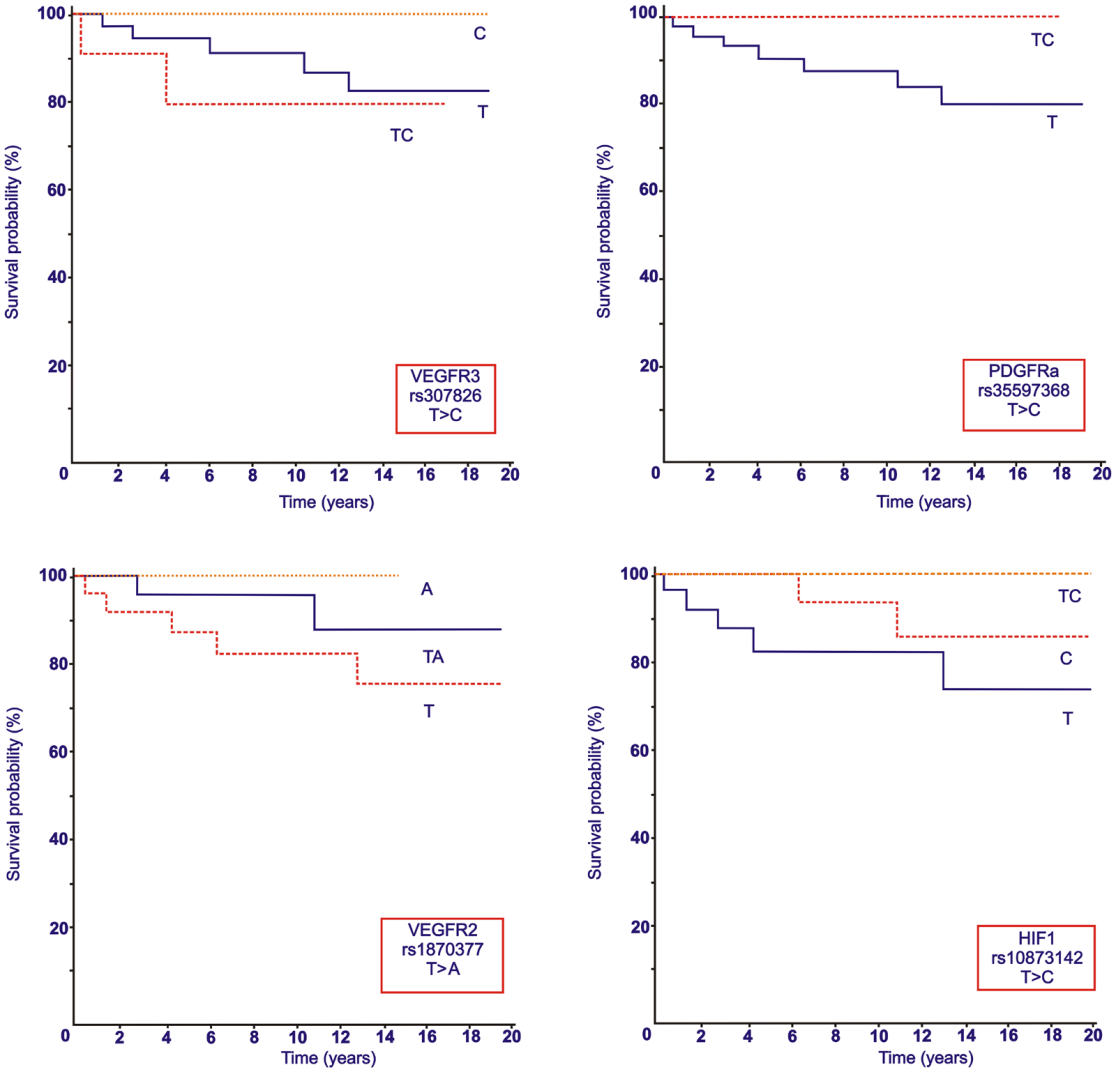
Overall Survival (OS, expressed in months) according to selected VEGFR-2, VEGFR-3, PDGFR-α and HIF1-α polymorphisms

We further investigated the prognostic role of SNPs on OS. As Figure 2 shows, the following polymorphisms were significantly correlated with a better OS, although they did not achieve median survival. Patients with VEGFR-3 rs307826 C genotype had significantly better OS than those with T and TC (*p* = 0.042). It was observed a correlation between VEGFR-2 rs1870377A genotype and OS compared to T and T/A, and also with PDGFR-α rs35597368T/C instead than C and T genotypes. Five HIF1-α polymorphisms were closely associated with longer OS: rs2301113C, rs2057482C/T, rs1951795C, rs11158358G/C and rs10873142T/C. Finally, patients with ERCC1 rs11615A genotype had longer OS compared to those with G and AG genotypes (*p* = 0.039).

### Results from computational analysis

To hypothesize the mechanisms underlying the association between SNPs and tumor histology or patient prognosis, we compared the molecular effects of the alleles of each SNP by different prediction tools.

The rs307821 SNP lies in the C-terminal region of VEGFR-3, corresponding to the cytoplasmic domain. This variation leads to the substitution of an arginine, a positively charged and polar aminoacid, with a leucine, an aliphatic and hydrophobic aminoacid. These aminoacids are poorly compatible and this change is disfavoured and can affect the protein structure and function. This hypothesis is supported by the aminoacidic conservation of the homologous sequences of this gene. Moreover, according to miRBase predictions, the G allele harbours target sites for miR-328–5p and miR-3960 microRNAs, but the T allele destroys these target sites. As a consequence, transcripts of the T allele could be not efficiently regulated, leading to an over-expression of VEGFR-3. We should take into account that, despite these predicted target sites are not in the 3′UTR but in a coding exon, they can modulate the transcripts anyway, as reported in previous studies [14, 15, 16]. Taken together, these data suggest that the T allele could increase the VEGFR-3 protein expression and tumor vascularisation. Actually, we found that T allele is correlated to thymic carcinomas, that are more vascularised than thymomas.

Concerning rs1870377 SNP, it causes the substitution of glutamine, a polar amino acid, with histidine, another polar amino acid, suggesting that this change should have neutral effect on protein structure. miRBase tool predicted no microRNA target sites in both alleles of this SNP. SpliceAid2 resource pointed out no alterations in splicing regulatory proteins lying on this SNP. RegRNA 2.0 did not detect changes between the two alleles. Therefore, based on actual knowledge, this SNP seems do not alter the splicing RNA process, post transcriptional regulation or protein structure and we could hypothesize that the influence on tumors is due to other SNPs linked to rs1870377.

As for the rs307826 SNP, it causes the substitution of threonine, a slightly polar and small amino acid, with alanine, a small and tiny amino acid. Thus, this change should have neutral effect on protein structure. miRBase tool predicted no microRNA target sites in both the alleles of this SNP. SpliceAid2 and RegRNA 2.0 did not detect changes between the two alleles. However it was shown by immunohistochemistry (IHC) that AA allele had larger VEGFR3 protein expression in clear-cell renal cell carcinoma [17]. We hypothesize that the G allele correlates with a better OS due to the association of this allele with a lower protein amount and, as a consequence, with a lower tumor vascularisation.

## DISCUSSION

VEGF and VEGFR SNPs have been associated with risk of several tumors, including breast [18], glioma [19], colorectal [20], lung [21], and oral cancer [22]. Nevertheless, the role of VEGF and VEGFR2–3 as well as of HIF-1α, PDGFR-α and ERCC1 SNPs in TETs risk and clinical features have not been investigated so far.

**Figure 3:**
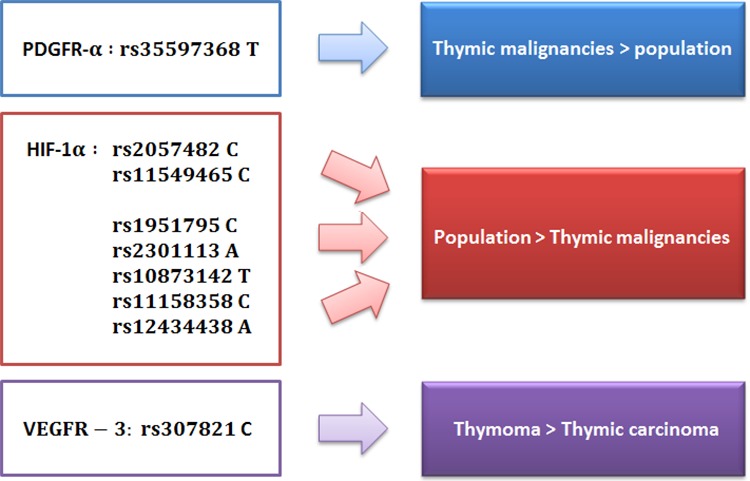
SNPs distribution in general population, thymomas and thymic carcinomas

In our study, we first analyzed the polymorphism frequencies in thymomas in comparison with general population. PDGFR-α rs35597368T polymorphism resulted significantly more frequent in thymomas than in general population (94.74% vs. 86.70%), thus suggesting that it may represent a risk factor for this disease (Figure 3).

On the other hand, the frequency of several HIF-1α polymorphisms resulted lower in the study cohort than in the general population, suggesting their protective role (Figure 3). Thus, HIF-1α rs11549465C polymorphism is more frequent in the general population than in our patients (92.50% vs. 85.09%). As for the rs11549465T allele, several studies have shown an association with cancer development, such as oral, prostatic, urinary, pancreatic and breast tumors. [23–30] In our study, rs11549465T frequency was higher in patients with TETs compared to general population (14.91 vs. 7.50%), although this difference was slightly significant (*p* = 0.07).

Also the frequency of HIF1-α rs2057482C SNP was lower in the cohort of patients with TETs (78.07% vs. 90.30%), as well as other HIF-1α SNPs (rs2301113A, rs10873142T, rs11158358C, rs12434438A, rs11549465C).

Furthermore, the frequency of VEGFR-3 rs307821 polymorphism was not different in our study cohort compared to general population, while it was higher in thymoma than in TC, and therefore it seems to correlate with a lower grade of malignancy (Figure 3). However, these indirect comparison with data from general population should be confirmed and validated in larger perspective trials.

We also investigated the prognostic role of several SNPs in patients with TETs. We found that VEGFR-2 rs1870377A polymorphism was correlated with longer OS. In the same view, VEGFR-2 rs1870377A polymorphism was associated with better prognosis in two studies regarding lymphoma diffuse large B-cell [31] and chronic myeloid leukemia with imatinib therapy. [32]

Conversely, in our study VEGFR-3 rs307826C SNP was correlated with longer OS, although it was previously associated with worse prognosis in patients with renal cell carcinoma treated with Sunitinib. [17, 33]

Moreover, ERCC1 rs11615A, PDGFR-α rs35597368 and several HIF-1 polymorphisms were also associated with longer OS.

In conclusion, the results of our study suggest that SNPs analysis may be useful in order to define high-risk patients after curative resection more likely to benefit from anti-angiogenic agents as adjuvant or successive line therapy at relapse or metastatic stages. Our results are even more interesting based on the preliminary results from a recent phase II study (NCT01621568) on the use of VEGFR tyrosine kinase inhibitor (TKI) sunitinib in patients with pretreated advanced thymic cancer. In this study, sunitinib demonstrated to be effective in patients with TETs, with 26% partial responses and 65% stable diseases in patients with TC (median OS = 16.3 months; median PFS = 6.7 months), while patients with thymomas showed 6% partial responses and 75% stable diseases (median OS = not reached; median PFS = 8.5 months). [34]

At present, another phase II study is evaluating bevacizumab in combination with anti-EGFR TKI erlotinib in patients with advanced thymoma and TC (NCT00369889).

To the best of our knowledge, there are no studies focusing on the correlation between the referred SNPs and thymic tumor risk and prognosis. Our results suggest, for the first time, that inherited abnormalities in PDGFR-α, HIF-1α and VEGFR-3 pathways influence the risk and aggressiveness of TETs. However, we recognize that our findings will require confirmation in perspective larger epidemiological studies and analyses focusing on the prognostic significance of SNPs in patients with TETs.

## MATERIALS AND METHODS

### Study population

The study population consisted of all consecutive patients aged 18 years or older who underwent surgery for TETs between 1993 and 2012. Other inclusion criteria included Eastern Cooperative Oncology Group (ECOG) performance status ≤ 2; adequate organ function; no serious concomitant disease. Written informed consent to undergo surgery was obtained from each subject and another consent for the biological procedures was obtained by alive patients. This study was carried out in accordance with the approval by the Ethical Committee of our Institution.

### SNP selection, DNA extraction, genotyping and predictions

SNPs in the above mentioned genes were selected using National Center for Biotechnology Information (NCBI) data, the Pupasuite software (http://pupasuite.bioinfo.cipf.es-version3.1) and reviewing medical literature, according to the following criteria:

- polymorphisms located in biologically relevant area of the gene (i.e. intron, 5′ UTR and 3′ UTR or promoter region)
- minor allele frequency (MAF) ≥ 10% (with the only exception of rs307821, rs11549465 and rs11549767)
- the genetic polymorphism was established and well documented.

Genomic DNA was extracted from paraffin-embedded tissue (30 mg) using the RecoverAll™ Total Nucleic Acid Isolation Kit for FFPE Tissues (Applied Biosystems, Foster City, CA, USA), according to the manufacturer's instructions.

Polymorphisms genotyping was performed using pre-designed TaqMan SNP Genotyping Assays (Applied Biosystems, Foster City, CA), according to the manufacturer's instructions. Amplifications and analysis were carried out on the 7300 Real-Time PCR System (Applied Biosystems), using the SDS software v1.4.0 for allelic discrimination (Applied Biosystems). About 10% of the samples were randomly remade for genotype confirmation and the results were 100% concordant. Data from general CEU population were provided by the HapMap project (http://www.HapMap.org). When these data were not available we considered the frequencies reported in the 1000 genome project (http://www.1000genomes.org).

We used different prediction tools in order to compare the molecular effects of the alleles of each SNP. miRBase (http://www.mirbase.org/) was used to detect putative target sites of microRNAs, while SpliceAid2 (http://www.introni.it/spliceaid. html) predicted binding sites of splicing regulatory proteins. As for RegRNA 2.0 (http://regrna2.mbc.nctu.edu.tw/), it found out regulatory RNA elements that could affect transcript maturation and translation.

### Statistical analysis

OS was defined as the interval between the date of surgery to death or last follow-up visit. OS was evaluated via the Kaplan-Meier method and Mantel-Haenszel log-rank test was employed to compare survival among groups. A Cox-regression model was applied to the data with a univariate approach and used to assess the role of polymorphisms as prognostic factors. All significance levels were set at a 0.05 value.

The genotype frequencies of VEGF-A, VEGFR2, VEGFR3, PDGFR-α, HIF1-α and ERCC1 were checked for the HWE and LD using Haploview, (Broad Institute, Cambridge, MA) to ensure that the markers were appropriate for inclusion in the haplotype estimates. The LD was measured by the disequilibrium coefficient (D), and LD significance was considered at a *D* ≥ 80%. The most common genotypes in control subjects were considered as references. Association between categorical variables was checked by using a chi-square test and a Fisher's exact probability test. The Benjamini-Hochberg correction method was used to adjust the values for multiple comparisons [35]. Statistical analysis was performed with MedCalc software version 10.4.8 for Windows.

### Summary sentence

A better understanding of the molecular biology of TETs represents a key challenge. The results of the study showed, for the first time, that polymorphisms (SNPs) of PDGFR-α, HIF-1α and VEGFR-3 influence the risk and aggressiveness of thymic tumors, suggesting that SNPs may be useful in order to define high-risk patients after curative resection.

## Abbreviations

TETs: Thymic epithelial tumors
TC: thymic carcinomas
VEGF-A: Vascular endothelial growth factor-A
VEGFR1: Vascular endothelial growth factor receptor 1
VEGFR2: Vascular endothelial growth factor receptor 2
KDR: kinase insert domain receptor
SNPs: single nucleotide polymorphisms
PDGF: Platelet-derived growth factor
PDGFRα: Platelet-derived growth factor receptor-α
HIF-1α: Hypoxia-inducible factor-1α
ERCC1: Excision repair cross-complementation group 1
ECOG: Eastern Cooperative Oncology Group
NCBI: National Center for Biotechnology Information
MAF: minor allele frequency
OS: Overall Survival
HWE: Hardy–Weinberg equilibrium
LD: linkage disequilibrium
TKI: tyrosine kinase inhibitor
